# Changes in Pepsinogen Activity in Biological Fluids of Pregnant Women with Newborns of Different Weights

**DOI:** 10.3390/biomedicines14061258

**Published:** 2026-05-31

**Authors:** Elena Kolodkina, Sergey Lytaev, Mikhail Galagudza

**Affiliations:** 1 Department of Normal Physiology, St. Petersburg State Pediatric Medical University, 194100 Saint Petersburg, Russia; 2 Almazov National Medical Research Center, 197341 St. Petersburg, Russia; galagudza@almazovcentre.ru

**Keywords:** pregnancy, enzyme level in the blood, pepsinogen, functional system mother–placenta–fetus, newborn

## Abstract

**Background**: The main “peptic” cells of the gastric glands provide the body’s only source of pepsinogen synthesis and incretion. Small amounts of endogenously supplied pepsinogen in biological fluids play an important role in anabolic processes in the mother and newborn. **The aim**: This work aimed to analyze the dynamics of pepsinogen activity in the blood serum, saliva, urine, and coprofiltrate in pregnant women in each trimester of pregnancy and in the postpartum period, taking into account the body weight of the newborn—normal weight, underweight, and overweight. **Methods**: Data from studies involving non-pregnant (n = 45) and pregnant (n = 152) women with newborns with different weights were analyzed. There were 86 women with a normal-weight newborn, 34 women with an underweight newborn, and 32 women with an overweight newborn. Total proteolytic activity in biological fluids was determined using the spectrophotometric tyrosine (tyr) Kunitz–Northrop method modified by Korot’ko. A 2% solution of dry plasma was used as a substrate. **Outcomes**: In non-pregnant women, the blood proteolytic activity was 58.1 ± 1.4 tyr U/mL, and saliva at 1520.9 ± 112.2 tyr U/mL, urine at 4520.3 ± 154.3 tyr U/mL, and coprofiltrate at 442.2 ± 20.5 tyr U/mL. We established that the pepsinogen activity during pregnancy is distributed unevenly, taking into account the body weight of the newborn, and changes significantly in women with an underweight or overweight newborn. **Conclusions**: Pepsinogen homeostasis in pregnant women is maintained by renal and extrarenal pathways, and an important role is played by the salivary glands, with the most significant changes occurring in women with overweight and underweight newborns.

## 1. Introduction

**Figure 1:**
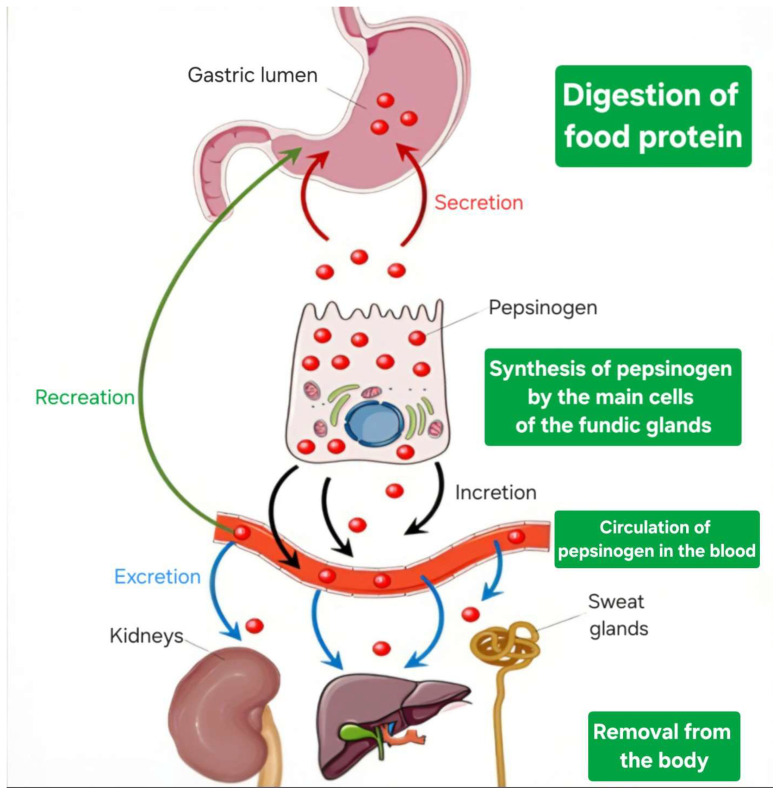
The relationship between the pathways and organs of secretion, incretion, and excretion of pepsinogen. Original figure.

According to the scientific literature, the level of pepsinogen in the blood is maintained through its intake via true incretion and the apoptosis, cytolysis, lysis, and necrosis of cells. Pepsinogen incretion occurs when the cells of the digestive glands secrete the inactive precursor of pepsin (pepsinogen) into the systemic bloodstream and lymphatic system. The enzyme is removed from the blood via the respiratory route and from the body via the excretory route. Part of pepsinogen is deposited in organs and in the blood itself, since erythrocytes and plasma proteins can sorb and desorb enzyme molecules. A certain amount of the enzyme binds to inhibitors via the vascular endothelium [1,2,3,4]. Thus, pepsinogen homeostasis is ensured in the body. Figure 1 shows the relationship between the distribution, synthesis, and excretion pathways of pepsinogen.

Pepsinogen (the precursor of pepsin), as a proteolytic enzyme, is synthesized by the main cells of the stomach. The main condition for synthesis is the acidic environment of the stomach. Through metabolism, pepsinogen enters the vascular system; from there, one part is returned back for resynthesis, and the other part is removed (excreted) from the body through renal and extrarenal pathways (Figure 1). Using metabolic data, researchers have described the dynamics of proteolytic activity in human biological fluids. Of particular interest in this regard is the information obtained from studying pregnant women and the “functional system mother–placenta–fetus” [5,6,7,8,9].

In summary, in the body, the main “peptic” cells of the gastric glands are the only source of the synthesis and incretion of pepsinogen. An analysis of the scientific literature has shown that the least studied mechanism is the distribution of pepsinogen in the “mother–fetus” and “lactating woman–newborn child” systems [10,11,12,13,14].

In the mother’s body, enzymes are increted from various digestive glands [15,16,17], while the stomach glands supply pepsinogen to the blood, which passes through the circulatory system to the fetus through the amnio-placental barrier. After birth, pepsinogen passes through colostrum and mother’s milk to the (breast-fed) newborn and is thus used for autolytic digestion [18]. Pepsinogen, secreted by the stomach, has regulatory and anabolic activity, the function of which changes by trimester during pregnancy [1,19,20,21].

Increted pepsinogen can stimulate anabolic processes and promote the synthesis of substances necessary for fetal growth and development due to its involvement in metabolism and nutrition (autotrophic, heterotrophic, trophoblastic, hematotrophic, amniotrophic, and lactotrophic types). Pepsinogen’s regulatory function is manifested in the interaction between the mother and fetus; increased pepsinogen secretion into the systemic circulation may indicate a functional need for interaction between the mother and fetus. Furthermore, pepsinogen can influence the secretory activity of the digestive glands, regulating the production of their own enzymes [1,19].

The level of the enzyme in the blood may be associated with changes in hydrostatic resistance during pregnancy. Renal and extrarenal pathways for the release of pepsinogen from the blood take part in maintaining homeostasis [3,10,11,22]. Extrarenal mechanisms include the secretion of the enzyme in saliva, sweat glands, and feces (coprofiltrate) [23,24]. While there are data on the processes of excretion and recretion of pepsinogen through measurement of its concentration/activity in urine and feces (coprofiltrate), as well as its secretion by the salivary glands [5,10], data over time by trimester of pregnancy and after childbirth have not been studied.

The novelty of this study lies in the investigation of pepsinogen in pregnant women with newborns of different birth weights. The results obtained complement the data on the distribution of the proteolytic activity of various biological fluids in these women. Hence, this is the first study to establish a relationship between the condition and maturity of the fetus and the characteristics of the proteolytic activity of biological fluids in pregnant women.

The purpose of this work was to study the activity of pepsinogen in biological fluids (blood, saliva, urine, and coprofiltrate) in pregnant women in the dynamics of pregnancy and in the postpartum period, taking into account the body weight of the newborn: normal weight, underweight, and overweight.

## 2. Materials and Methods

The study of biological fluids was carried out in non-pregnant (45 women in the control group) and pregnant (152) women under the pregnancy monitoring program. All pregnant women studied were citizens of the Russian Federation who attended public healthcare facilities and had no dietary restrictions. We excluded pregnant women with a history of gastrointestinal diseases, pregnancy complications, taking medications during pregnancy, and those with co-morbidities such as smoking and alcohol consumption. The pregnant women had newborns with different body weights (86 normal weight, 34 underweight, and 32 overweight).

The average birth weight of normal-weight infants was 3367.5 ± 75.1 g, which corresponded to normal values, with an average Apgar score of 7.8 ± 0.3 at the first minute and 8.4 ± 0.4 at the fifth minute. The newborns’ average body length was 50.3 ± 4.7 cm, and their average head circumference was 34.2 ± 2.8 cm. The heart rate in the first days of life averaged 129.4 ± 10.8 beats per minute.

The average birth weight of underweight newborns was 2228.2 ± 64.1 g, significantly below normal values, with an average Apgar score of 6.4 ± 0.2 at the first minute and 7.2 ± 0.3 at the fifth minute. The body length of these newborns averaged 37.4 ± 3.2 cm, significantly lower than that of infants with a normal body weight. The head circumference averaged 28.1 ± 1.8 cm. The average heart rate in the first days of life was 124.1 ± 11.2 beats per minute, which was also significantly lower than that of infants with normal body weight.

The average birth weight of overweight newborns was 4105.1 ± 86.2 g, which is above normal, with an average Apgar score of 7.2 ± 0.3 points at the first minute and 8.1 ± 0.4 points at the fifth minute. The average body length of newborns was 55.8 ± 5.1 cm, and the average head circumference was 38.4 ± 3.1 cm. The average heart rate in the first days of life was 123.7 ± 10.7 beats per minute.

This study was conducted on women in the St. Petersburg Snegirev’s Maternity Hospital No. 6. All women (the control and research groups) were informed about the purpose and methods and gave written voluntary informed consent to participate in the study (protocol no. 0608-23 dated 7 August 2023, of the Local Ethics Committee of the Almazov National Medical Research Center).

The content and activity of pepsinogen in the serum of peripheral blood, saliva, urine, and coprofiltrate were assessed in the control group and pregnant women by trimester of pregnancy and in the postpartum period. The gestational age at the time of delivery was determined by the date of the last menstrual period, the date of the first fetal movement, the visit to the antenatal clinic, and data from the external obstetric and ultrasound research. Clinical data on the course of pregnancy and childbirth were obtained from the exchange and notification observation card of the pregnant and postpartum women, the birth history, and the developmental history of the newborn.

The total proteolytic activity of biological fluids was determined at the optimal pH level for pepsin action using an SF-46 spectrophotometer (Russian Federation) at a wavelength of 280 nm. The quantitative determination of pepsinogen in blood plasma, urine, saliva, and coprofiltrate was determined by the proteolytic activity at pH 1.5–2.0 with spectrophotometric determination of tyrosine using the Kunitz–Northrop method modified by Korot’ko [25,26] via the incubation of the protein substrate with the studied biological fluids (blood serum, urine, saliva, and coprofiltrate).

The level of enzymatic activity was determined according to the amount of tyrosine formed during proteolysis. A 2% solution of lyophilized blood plasma was used as a substrate, incubated with the protein at 37 °C for 24 h at pH 1.5–2.0. Other proteases contained in saliva, urine, or coprofiltrate could not influence the results, since the enzyme activity was assessed within the narrow pH range of 1.5–2.0.

The enzyme activity was calculated using a calibration curve of standard tyrosine solutions. One unit of pepsinogen activity in 1 mL of biological fluid was defined as the amount of enzyme catalyzing the formation of 1 μg of tyrosine during the enzymatic hydrolysis of the protein substrate at 37 °C.

## 3. Results

The results show that, in non-pregnant women, the proteolytic activity of the blood was 58.1 ± 1.4 tyr U/mL, with saliva at 1520.9 ± 112.2 tyr U/mL, urine at 4520.3 ± 154.3 tyr U/mL, and coprofiltrate at 442.2 ± 20.5 tyr U/mL. The proteolytic activity of the blood in pregnant women with a normal-weight newborn was characterized by lower values compared with non-pregnant women throughout all trimesters of pregnancy and in the postpartum period (Table 1). The revealed dynamics reflect the restructuring of enzymatic homeostasis in the “mother–fetus” system and may be associated with the physiological adaptation of the pregnant woman’s body, including changes in hormonal status and the redistribution of metabolic flows.

In pregnant women with an underweight newborn, a significant increase in blood pepsinogen activity was observed in the third trimester, which sharply decreased after delivery. In pregnant women with an overweight newborn, the serum pepsinogen activity increased throughout pregnancy, reaching maximum values in the third trimester of pregnancy, with a subsequent decrease in the postpartum period. Significant differences were found in the serum enzyme activity values in pregnant women with an underweight newborn in the third trimester (*p* < 0.001) and in pregnant women with an overweight newborn in the second (*p* < 0.05) and third (*p* < 0.001) trimesters of pregnancy compared with pregnant women with a normal-weight newborn (Table 1).

Salivary glands are known to play a role in homeostasis through their secretory mechanism for releasing pepsinogen from the blood. The pepsinase activity in the saliva collected from women at various stages of pregnancy varied significantly, increasing from trimester to trimester and approaching that of non-pregnant women after delivery (Table 2). In pregnant women with a normal-weight newborn, the salivary proteolytic activity was lower than in the controls in the first trimester of pregnancy but subsequently increased throughout pregnancy, reaching maximum values by the end of pregnancy (1.7 times higher than similar control values; *p* < 0.05). In the postpartum period, the enzyme excretion with saliva decreased and became the same as the control. In pregnant women with an underweight newborn, the salivary pepsinogen activity increased beginning in the second trimester of pregnancy, decreasing after delivery to levels seen in the control group. In pregnant women with an overweight newborn, higher salivary enzyme levels were observed from the very beginning of pregnancy compared with non-pregnant women, reaching maximum values at the end of pregnancy and remaining at high levels in the postpartum period. Thus, salivary pepsinogen activity reflects enzyme incretion processes and can serve as one of the indicators used in saliva diagnostics [21].

The urinary enzyme excretion varied between pregnant women with newborns with different weights. In pregnant women with normal-weight newborns, the pepsinogen activity was higher than the control values throughout pregnancy but decreased by 2.7 times (*p* < 0.001) in the postpartum period compared with the third trimester (Table 3).

In pregnant women with underweight newborns, the urinary enzyme excretion was significantly higher than in women with normal-weight newborns (*p* < 0.05). After delivery, the urinary proteolytic activity levels decreased significantly, reaching levels similar to those in non-pregnant women. In pregnant women with overweight newborns, the renal pepsinogen excretion remained high throughout pregnancy, decreasing in the postpartum period. This can likely be explained by the way pepsinogen homeostasis is maintained in the pregnant woman’s body to support anabolic processes in the fetus and newborn.

Data on the proteolytic activity of the coprofiltrate showed a reverse trend in enzyme excretion compared with urine. Pepsinogen activity in the coprofiltrate (1:4 dilution) was higher in non-pregnant women; it decreased from the first to the third trimester of pregnancy and then increased slightly in the postpartum period in all groups of women studied (Table 4). Thus, a small amount of pepsinogen is excreted in feces; its excretion level increases after childbirth but remains lower than that of non-pregnant women.

**Figure 2:**
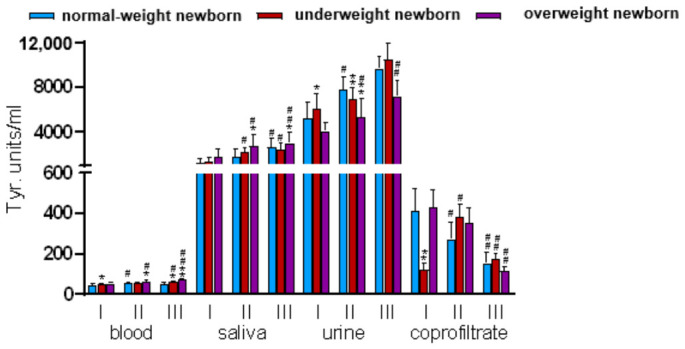
Changes in the proteolytic activity of biological fluids in pregnant women with newborns with different body weights. (Note: Significance of differences in indicators in pregnant women with normal newborn body weight: *—*p* < 0.05; **—*p* < 0.001; significance of differences with indicators in pregnant women in the first trimester: #—*p* < 0.05; ##—*p* < 0.001.

A comparative analysis of changes in the pepsinogen activity in various biological fluids during pregnancy compared with non-pregnant women revealed the following results (Figure 2).

By the end of pregnancy, the blood proteolytic activity approached that of non-pregnant women, especially in women with underweight newborns. In pregnant women with normal-weight newborns, the blood pepsinogen activity levels remained below the control values throughout pregnancy. The same dynamics were observed for salivary pepsinogen activity, but its levels were 1.2–1.6 times higher (*p* < 0.001) in all pregnant women in the latter stages of pregnancy, reflecting the involvement of resective processes in pepsinogen homeostasis.

The kidneys are most actively involved in maintaining this enzyme. In all the pregnant women studied, the uropepsinogen levels in the last trimester were more than twice as high as those in non-pregnant women. In the fecal filtrate, a decrease in proteolytic activity was mainly observed at low pH values, reaching below 50% of the baseline values at the end of pregnancy, indicating enzyme retention in the maternal body. Thus, we have discovered the constancy of pepsinogen in the blood plasma of pregnant women, which is variable in biological fluids, taking into account the body weight of the newborn.

## 4. Discussion

Pepsinogen activity in blood plasma depends on the amount of enzyme incretion in the stomach, where the main glandulocytes synthesize it with the main secretion into the lumen of the glands and a partial secretion into the blood and lymph nodes in a ratio of 100: 1 and 1000: 1, respectively [1,4,11]. Such small amounts of endogenously supplied pepsinogen play an important role in anabolic processes in the mother’s body and in the newborn [6,18,27]. Increted pepsinogen affects protein metabolism, increasing the incorporation of amino acids into the synthesized protein, including in the growing body of the fetus [5,10,28]. Therefore, the constancy of the enzyme in the mother’s body is genetically predetermined by the connection with the fetus and newborn.

The release of increted pepsinogen from the blood (recretion) suggests the possibility of its participation in physiological processes, and the irreversible loss of the enzyme from the body through excretion (kidneys, sweat, lacrimal glands, terminal sections of the intestine) reduces the level of amino acid metabolism [2,3,10]. The retention of pepsinogen in the body is due to its deposition in the organs storing blood; its circulation in the blood; its degradation into peptides and amino acids; and its utilization, anabolization, fixation on the endothelium for the transport of metabolites [10,15].

We studied the pepsinogen activity in non-pregnant and pregnant women, according to their newborn’s body weight. The homeostasis of pepsinogen metabolism in pregnant and non-pregnant women is maintained by renal and extrarenal pathways. Among these pathways, the salivary glands play an important role, secreting the enzyme from the blood for its participation in hydrolytic processes in gastric contents, along with pepsin.

Subsequent plasma pepsinogen metabolism depends on urinary pepsinogen excretion, which increased from trimester to trimester and decreased sharply after childbirth. Furthermore, saliva in the second and third trimesters of pregnancy possessed higher proteolytic activity than that in the control group. Its levels decreased in the postpartum period, consistent with the dynamics of the protein-producing function in the mother–fetus system [10,29].

The excretion of pepsinogen in saliva is used as a diagnostic of the state of the digestive glands, which is as a probe-free method for assessing the enzyme-forming and excretory activity of the gastrointestinal tract and homeostatic parameters of the body [30,31]. Pepsinogen is mainly retained and utilized in the body; the unused part is excreted in feces and urine. According to the available data, a significant amount of pepsinogen is excreted by the kidneys, while other pathways of excretion and degradation of the enzyme are much lower in volume [2,10]. Our results showed that the proteolytic activity of urine steadily increases towards the end of pregnancy, which is associated with the increased excretion of the enzyme in urine and saliva.

The excretory–recretory origin of the proteolytic activity of the fecal coprofiltrate is explained by the detection of pepsinogen in feces. At the end of pregnancy, the enzyme content in the fecal coprofiltrate decreases sharply to preserve its activity, with the largest changes observed in pregnant women with overweight newborns. In the postpartum period, intestinal enzyme homeostasis is maintained for subsequent use in the relationship between the nursing mother and her child. Thus, studying the pepsinogen activity over time expands our understanding of the distribution of proteolytic activity in various biofluids in pregnant women with newborns of varying weights.

The excretion of proteolytic enzymes varies in different biological environments, sometimes even in opposite directions. Pepsinogen activity also varies depending on the birth weight of the newborn. In normal-weight newborns, enzyme activity in pregnant women remained consistently high throughout pregnancy, but significantly decreased in the postpartum period compared to the third trimester. In low-weight newborns, urinary enzyme concentrations were significantly higher than in the previous group. After delivery, protease activity levels dropped significantly, becoming similar to those in non-pregnant women. In overweight newborns, urinary pepsinogen levels remained high throughout pregnancy and, as in the previous groups, decreased in the postpartum period. We associate all of this with mechanisms for maintaining pepsinogen homeostasis in the pregnant woman’s body to ensure anabolic processes in the fetus and newborn.

The proteolytic activity of the fetal filtrate was fundamentally different from that of urine and showed an inverse relationship. Overall, pepsinogen activity in this environment was highest in the absence of pregnancy. Enzyme activity decreased in pregnant women from the first to the third trimester, after which it increased slightly in the postpartum period in all studied groups. Thus, the general trend for pregnant women regarding pepsinogen activity in the coprofiltrate is as follows: a small amount is removed, and its excretion rate increases after childbirth, but remains lower than in non-pregnant women.

In general, the urinary system is more active in metabolizing proteolytic enzymes. In all studied groups of pregnant women, uropepsinogen levels reached their maximum values in the last trimester and were significantly higher compared to non-pregnant women. In the gastrointestinal tract (coprofiltrate), a reduction in proteolytic enzyme activity was recorded only at lower pH values (high acidity). Subsequently, by the end of pregnancy, enzyme activity dropped to almost half of its baseline values, indicating enzyme retention in the mother’s body. Thus, we recorded a relative constancy of pepsinogen in the blood plasma of pregnant women, which dynamically varies in biological environments taking into account the body weight of the newborn.

## 5. Conclusions

This study assessed the proteolytic activity of the gastric chief cell enzyme in various biological environments of pregnant women over the course of each trimester and after delivery, according to the fetal weight parameters. The following trends were noted.

In pregnancies with underweight newborns, a significant increase in blood pepsinogen activity was observed only at the end of pregnancy, and the activity declined after delivery. In pregnancies with overweight newborns, the serum pepsinogen activity increased throughout pregnancy, reaching peak values in the third trimester, followed by a decline in the postpartum period.

The pepsinase activity in saliva collected from women at different stages of pregnancy varied significantly, increasing from trimester to trimester and approaching levels in non-pregnant women after delivery.

The urinary enzyme excretion varied between pregnant women with newborns with different weights. For example, in pregnant women with normal-weight fetuses, the pepsinogen activity was higher than the control values throughout pregnancy but decreased in the postpartum period compared with the third trimester.

The proteolytic activity of the fecal filtrate showed the opposite trend in enzyme excretion compared with urine. The pepsinogen activity in the fecal filtrate was higher in non-pregnant women; it decreased from the first to the third trimester of pregnancy and then increased slightly during the postpartum period in all groups of pregnant women studied.

## Figures and Tables

**Table 1:** Proteolytic activity of blood in women (tyr U/mL) depending on the body weight of the newborn.

Research Group of Pregnant Women	Trimester I	Trimester II	Trimester III	After Childbirth
Normal-weight newborn, n = 86	44.22 ± 3.34	53.81 ± 4.12	48.15 ± 1.30	44.39 ± 1.19
Underweight newborn, n = 34	48.31 ± 0.93	55.02 ± 1.07	62.33 ± 1.19 **	39.44 ± 1.06
Overweight newborn, n = 32	52.41 ± 1.28 *	64.43 ± 1.44 *	71.66 ± 1.33 *	60.07 ± 1.41 *

Note: Differences compared with values in pregnant women with a normal-weight newborn: *—*p* < 0.001; **—*p* < 0.05.

**Table 2:** Proteolytic activity of saliva in women (tyr U/mL) depending on the body weight of the newborn.

Research Group of Pregnant Women	Trimester I	Trimester II	Trimester III	After Childbirth
Normal-weight newborn, n = 86	1208.6 ± 48.2	1807.0 ± 75.6	2612.9 ± 93.3	1463.3 ± 53.8
Underweight newborn, n = 34	1316.3 ± 64.1	2167.2 ± 78.3	2443.5 ± 97.7 *	1553.8 ± 72.2
Overweight newborn, n = 32	1800.2 ± 182.5	2706.1 ± 215.8 **	3406.5 ± 263.6 **	2606.3 ± 193.8 **

Note: See Table 1. *—*p* < 0.001; **—*p* < 0.05.

**Table 3:** Renal pepsinogen secretion in women (tyr U/mL) depending on the body weight of the newborn.

Research Group of Pregnant Women	Trimester I	Trimester II	Trimester III	After Childbirth
Normal-weight newborn, n = 86	5200.8 ± 122.4	7800.1 ± 126.8	9650.1 ± 131.1	3698.5 ± 113.9
Underweight newborn, n = 34	6100.2 ± 186.1 **	6900.5 ± 204.1 **	10422.1 ± 258.8 **	4302.2 ± 157.4
Overweight newborn, n = 32	4100.6 ± 107.5 **	5300.3 ± 184.3 *	7270.8 ± 277.7 **	3937.9 ± 101.4

Note: See Table 1. *—*p* < 0.001; **—*p* < 0.05.

**Table 4:** Proteolytic activity of coprofiltrate in women (tyr U/mL) depending on the body weight of the newborn.

Research Group of Pregnant Women	Trimester I	Trimester II	Trimester III	After Childbirth
Normal-weight newborn, n = 86	410.2 ± 27.1	270.8 ± 20.4	153.8 ± 5.8	315.3 ± 14.5
Underweight newborn, n = 34	120.6 ± 7.8 *	381.6 ± 15.6 **	174.7 ± 9.2	342.5 ± 12.8
Overweight newborn, n = 32	430.4 ± 31.2	352.6 ± 16.4 **	110.3 ± 3.3	192.9 ± 8.5 *

Note: See Table 1. *—*p* < 0.001; **—*p* < 0.05.

## Data Availability

The data presented in this study are available on request from the corresponding author due to privacy and ethical restrictions.

## References

[1] Muller M.J., Defize J., Hunt R.H. (2020). Control of pepsinogen synthesis and secretion. Gastrointest. Endosc. Clin. N. Am..

[2] Korot’ko G.F. (2022). Recirculation and adaptation of enzymes of digestive glands secretion. Exp. Clin. Gastroent..

[3] Lushchak V.I. (2025). Symphony of Digestion: Coordinated Host–Microbiome Enzymatic Interplay in Gut Ecosystem. Biomolecules.

[4] Varro V. (2015). On the value of plasma and urinary pepsinogen determinations. J. Indian Med. Profess..

[5] Kolodkina E.V., Lytaev S.A. (2024). Study of the activity of digestive enzymes in biological fluids in women during pregnancy. Russ. Biomed. Res..

[6] King J.C. (2018). Physiology of pregnancy and nutrient metabolism. Am. J. Clin. Nutr..

[7] Borowitz D. (2025). Non-Pancreatic Digestive Enzymes. Biomolecules.

[8] Litwack G. (2018). Human Biochemistry.

[9] Morton A. (2024). Investigating gastrointestinal disorders in pregnancy. Obstet. Med..

[10] Korot’ko G.F. (2003). Rekretsiia fermrntov i gormonov ékzokrinnymi zhelezami [Recretion of enzymes and hormones by exocrine glands]. Usp. Fiziol. Nauk.

[11] Korot’ko G.F. (2015). Endocrecretion of enzymes in modulation of digestive glands activity. Rus. J. Gastroent. Hep. Proct..

[12] Chandra M., Paray A.A. (2024). Natural Physiological Changes during Pregnancy. Yale J. Biol. Med..

[13] Kothari S., Afshar Y., Friedman L.S., Ahn J. (2024). AGA Clinical Practice Update on Pregnancy-Related Gastrointestinal and Liver Disease: Expert Review. Gastroenterology.

[14] Kazma J.M., Anker J., Allegaert K., Dallmann A. (2020). Anatomical and physiological alterations of pregnancy. J. Pharmacokinet. Pharmacodyn..

[15] Qin Y., Geng J.X., Huang B. (2023). Clinical value of serum pepsinogen in the diagnosis and treatment of gastric diseases. World J. Gastrointest. Oncol..

[16] Korot’ko G.F. (2014). The role of the salivary glands in maintaining the relative constancy of blood hydrolytic activity. Russ. Physiol. J. I.M. Sechenov..

[17] Richter C., Tanaka T., Yada R.Y. (2018). Mechanism of activation of the gastric aspartic proteinases: Pepsinogen, progastricsin and prochymosin. Biochem. J..

[18] Kolodkina E.V., Lytaev S.A., Galagudza M.M. (2024). Indicators of the activity of digestive enzymes and transaminases in saliva and coprofiltrate in women during pregnancy. Russ. Biomed. Res..

[19] Ayala N.K., Fain A.C., Cersonsky T.E.K. (2023). Early pregnancy dispositional optimism and pregnancy outcomes among nulliparous people. Am. J. Obstet. Gynecol. MFM.

[20] Nguyen C.L., Dao T.T., Phi T.N., Nguyen T.P. (2022). Serum pepsinogen: A potential non-invasive screening method for moderate and severe atrophic gastritis among an asian population. Ann. Med. Surg..

[21] Ying E., Qian Y., Tao S., Hang X., Xue-Rong Z., Hua-Chuan Z. (2023). The relationship between pepsinogen C and gastric carcinogenesis: A transgene and population study. BMC Cancer.

[22] Blandizzi C., Colucci R., Carignani D. (2021). Positive Modulation of Pepsinogen Secretion by Gastric Acidity After Vagal Cholinergic Stimulation. Am. J. Physiol..

[23] Kolodkina E., Lytaev S. (2025). The Intestinal Mechanisms in the Excretion of Pepsinogen, Amylase and Lipase in Coprofiltrate in Women During Pregnancy and the Postpartum Period. Biomolecules.

[24] Mandel I.D. (2019). The role of saliva in maintaining oral homeostasis. Am. Dent. Assoc..

[25] Ivanov D.O., Avrel’kina E.V., Aleksandrovich Y.S., Aleshina E.I. (2019). Rukovodstvo po Perinatologii.

[26] Dewi M., Andarwulan N., Wahyuningsih U., Kazimierczak R., Średnicka-Tober D. (2025). Maternal Long-Chain Polyunsaturated Fatty Acids Status in Pregnancy and Newborn Body Composition. Nutrients.

[27] Lee S.Y. (2016). Endoscopic gastritis, serum pepsinogen assay, and Helicobacter pylori infection. Korean J. Intern. Med..

[28] Boroumand M., Olianas A., Cabras T. (2021). Saliva, a bodily fluid with recognized and potential diagnostic applications. J. Sep. Sci..

[29] Hofman L.F. (2017). Human Saliva as a Diagnostic Specimen. Am. Soc. Nutr. Sci..

[30] Kaufman E., Lamster I.B. (2016). The diagnostic applications of saliva—A review. Crit. Rev. Oral Biol. Med..

[31] Streckfus C.F., Bigler L.R. (2012). Saliva as a diagnostic fluid. Salivary Glands and Saliva. Oral Dis..

