# Correction: Kolodkina et al. Changes in Pepsinogen Activity in Biological Fluids of Pregnant Women with Newborns of Different Weights. *Biomedicines* 2026, *14*, 1258

**DOI:** 10.3390/biomedicines14071498

**Published:** 2026-07-02

**Authors:** Elena Kolodkina, Sergey Lytaev, Mikhail Galagudza

**Affiliations:** 1 Department of Normal Physiology, St. Petersburg State Pediatric Medical University, 194100 Saint Petersburg, Russia; 2 Almazov National Medical Research Center, 197341 St. Petersburg, Russia; galagudza@almazovcentre.ru

**Text Correction**

There was an error in the original publication [1]. Authors’ revisions made during the proofreading stage were inadvertently omitted due to an internal oversight. A correction has been made to the Discussion, Paragraph Numbers 7, 8, 9, 10:

The excretion of proteolytic enzymes varies in different biological environments, sometimes even in opposite directions. Pepsinogen activity also varies depending on the birth weight of the newborn. In normal-weight newborns, enzyme activity in pregnant women remained consistently high throughout pregnancy, but significantly decreased in the postpartum period compared to the third trimester. In low-weight newborns, urinary enzyme concentrations were significantly higher than in the previous group. After delivery, protease activity levels dropped significantly, becoming similar to those in non-pregnant women. In overweight newborns, urinary pepsinogen levels remained high throughout pregnancy and, as in the previous groups, decreased in the postpartum period. We associate all of this with mechanisms for maintaining pepsinogen homeostasis in the pregnant woman’s body to ensure anabolic processes in the fetus and newborn.

The proteolytic activity of the fetal filtrate was fundamentally different from that of urine and showed an inverse relationship. Overall, pepsinogen activity in this environment was highest in the absence of pregnancy. Enzyme activity decreased in pregnant women from the first to the third trimester, after which it increased slightly in the postpartum period in all studied groups. Thus, the general trend for pregnant women regarding pepsinogen activity in the coprofiltrate is as follows: a small amount is removed, and its excretion rate increases after childbirth, but remains lower than in non-pregnant women.

In general, the urinary system is more active in metabolizing proteolytic enzymes. In all studied groups of pregnant women, uropepsinogen levels reached their maximum values in the last trimester and were significantly higher compared to non-pregnant women. In the gastrointestinal tract (coprofiltrate), a reduction in proteolytic enzyme activity was recorded only at lower pH values (high acidity). Subsequently, by the end of pregnancy, enzyme activity dropped to almost half of its baseline values, indicating enzyme retention in the mother’s body. Thus, we recorded a relative constancy of pepsinogen in the blood plasma of pregnant women, which dynamically varies in biological environments taking into account the body weight of the newborn.

**Data Availability Statement Correction**

There was an error in the original publication [1]. Authors’ revisions made during the proofreading stage were inadvertently omitted due to an internal oversight. The correct Data Availability Statement is as follows: The data presented in this study are available on request from the corresponding author due to privacy and ethical restrictions.

The authors state that the scientific conclusions are unaffected. This correction was approved by the Academic Editor. The original publication has also been updated.
